# Association of common candidate variants with vascular malformations and intracranial hemorrhage in hereditary hemorrhagic telangiectasia

**DOI:** 10.1002/mgg3.377

**Published:** 2018-03-06

**Authors:** Ludmila Pawlikowska, Jeffrey Nelson, Diana E. Guo, Charles E. McCulloch, Michael T. Lawton, Helen Kim, Marie E. Faughnan, the Brain Vascular Malformation Consortium HHT Investigator Group, Murali Chakinala, Marianne Clancy, James R Gossage, Katharine Henderson, Steven W Hetts, Vivek Iyer, Raj Kasthuri, Timo Krings, Doris Lin, Johannes Jurgen Mager, Douglas Marchuk, Justin P McWilliams, Jamie McDonald, Jeffrey Pollak, Felix Ratjen, Karen Swanson, Karel terBrugge, Dilini Vethanayagam, Andrew White, Pearce Wilcox

**Affiliations:** ^1^ Center for Cerebrovascular Research Department of Anesthesia and Perioperative Care University of California San Francisco CA USA; ^2^ Institute for Human Genetics University of California San Francisco CA USA; ^3^ Department of Epidemiology and Biostatistics University of California San Francisco CA USA; ^4^ Department of Neurosurgery Barrow Neurological Institute Phoenix AZ USA; ^5^ Division of Respirology Department of Medicine and Li Ka Shing Knowledge Institute St. Michael's Hospital Toronto ON Canada; ^6^ Division of Respirology Department of Medicine University of Toronto Toronto ON Canada

**Keywords:** arteriovenous malformation, genetic modifiers, hereditary hemorrhagic telangiectasia, intracerebral hemorrhage, vascular malformation

## Abstract

**Background:**

Hereditary hemorrhagic telangiectasia (HHT) is caused by mutations in TGFβ/BMP9 pathway genes and characterized by vascular malformations (VM) including arteriovenous malformations (AVM) in lung, liver, and brain, which lead to severe complications including intracranial hemorrhage (ICH) from brain VM. The clinical heterogeneity of HHT suggests a role for genetic modifier effects. Common variants in loci that modify phenotype severity in *Tgfb* knockout mice were previously reported as associated with lung AVM in HHT. Common variants in candidate genes were reported as associated with sporadic brain AVM and/or ICH. We investigated whether these variants are associated with HHT organ VM or with ICH from brain VM in 752 Caucasian HHT patients enrolled by the Brian Vascular Malformation Consortium.

**Methods:**

We genotyped 11 candidate variants: four variants reported as associated with lung AVM in HHT (*PTPN14* rs2936018, *USH2A* rs700024, *ADAM17* rs12474540, rs10495565), and seven variants reported as associated with sporadic BAVM or ICH (APOE ε2, *ANGPTL4* rs11672433, *EPHB4* rs314308, *IL6* rs1800795, *IL1B* rs1143627, *ITGB8* rs10486391, *TNFA* rs361525). Association of genotype with any VM, lung AVM, liver VM, brain VM or brain VM ICH was evaluated by multivariate logistic regression adjusted for age, gender, and family clustering.

**Results:**

None of the 11 variants was significantly associated with any phenotype. There was a trend toward association of *USH2A* rs700024 with ICH (OR = 2.77, 95% CI = 1.13–6.80, *p* = .026).

**Conclusion:**

We did not replicate previously reported associations with HHT lung AVM and variants in *Tgfb* modifier loci. We also did not find significant associations between variants reported in sporadic brain AVM and VM or ICH in HHT.

## 1 INTRODUCTION

Hereditary hemorrhagic telangiectasia (HHT) is caused by mutations in TGFβ/BMP9 pathway genes, most commonly *ENG* (OMIM 131195) or *ACVRL1 (ALK1*, OMIM 601284). HHT is characterized by vascular malformations (VM) ranging from small skin and mucosal telangiectases to arteriovenous malformations (AVM) in lung, liver, and brain, which can lead to severe complications including intracranial hemorrhage (ICH) from ruptured brain VM. The clinical heterogeneity of HHT suggests a potential role for genetic modifier effects. Identification and validation of such modifiers could inform disease prognosis and guide clinical management of HHT patients and provide potentially useful stratifier biomarkers for clinical trials. A few such genetic associations with HHT disease features have been reported. Common variants in two loci that modify phenotype severity in *Tgfb* knockout mice (TGFβ modifier loci) have been associated with lung AVM in HHT families (Benzinou et al., 2012; Kawasaki et al., 2014). We previously showed that the *ACVRL1* c.314‐35A>G polymorphism, associated with sporadic brain AVM (Pawlikowska et al., 2005; Simon et al., 2006), is also associated with VM in HHT, but only among patients with *ENG* mutations (Pawlikowska et al., 2015). Common variants in other candidate genes have previously been reported to be associated with sporadic BAVM (Kim et al., 2009; Mikhak et al., 2011; Pawlikowska et al., 2005; Su et al., 2010) or with sporadic BAVM ICH (Achrol et al., 2006, 2007; Hysi et al., 2007; Pawlikowska et al., 2004, 2006; Weinsheimer et al., 2009). We investigated whether the four TGFβ modifier locus variants previously reported in HHT lung AVM and seven variants from sporadic AVM studies are associated with organ VM and with brain VM ICH in a large cohort of Caucasian HHT patients enrolled by the Brain Vascular Malformation Consortium (BVMC) (Akers et al., 2013).

## 2 MATERIALS AND METHODS

### 2.1 Ethical compliance

The study protocol was approved by the institutional review board at each recruiting center. All patients provided written informed consent for genetic studies and a blood or saliva sample for DNA extraction.

### 2.2 Cohort

The study includes 752 Caucasian HHT patients enrolled by the BVMC at multiple recruiting centers in the US, Canada, and the Netherlands between 2010 and 2015. Cohort recruitment has been previously described (Akers et al., 2013; Pawlikowska et al., 2015). Patients were screened for organ VM and other clinical features according to standard clinical practice and International HHT Guidelines (Faughnan et al., 2011), including: comprehensive history, physical, routine blood tests, and clinical screening for recurrent spontaneous epistaxis and HHT‐related gastrointestinal bleeding. All patients were screened for pulmonary AVM by contrast echocardiography or by chest CT. All patients were screened for brain VM by magnetic resonance imaging. If screening was positive for pulmonary AVM or brain VM (Krings et al., 2015), patients underwent further diagnostic imaging and treatment, where appropriate. For liver VM, all patients underwent clinical screening (chronic right upper quadrant pain, portal hypertension, high‐output heart failure, liver bruit on examination, abnormal liver function tests) as well as echocardiography for high‐output cardiopathy. If screening was positive, then diagnostic liver imaging was performed (ultrasound Doppler, contrast CT or contrast MRI) and therapy recommended where appropriate. The BVMC HHT cohort targets 25% brain VM‐positive patients; other characteristics are similar to other cohorts (Letteboer et al., 2006; Nishida et al., 2012). Brain VM ICH included any ICH during HHT disease course (prior to enrollment or during follow‐up, including after brain VM treatment). For lung AVM, we also defined a severe subphenotype as: diffuse lung AVM and/or feeding artery diameter of largest lung AVM ≥5 mm and/or presence of any of the following complications: ischemic stroke, brain abscess, massive hemoptysis, spontaneous hemothorax.

### 2.3 Genotyping

DNA was extracted at the NINDS Repository at Coriell Institute (http://ccr.coriell.org/Sections/Collections/NINDS), or at UCSF (saliva). We genotyped 11 candidate variants including seven variants from sporadic BAVM studies (*APOE* ε2 haplotype comprising rs7412 and rs429358 (Achrol et al., 2007; Pawlikowska et al., 2006), *ANGPTL4* rs11672433 (Mikhak et al., 2011), *EPHB4* rs314308 (Weinsheimer et al., 2009), *IL6*‐174G>C rs1800795 (Kim et al., 2008; Pawlikowska et al., 2004), *IL1B*‐31T>C rs1143627 (Kim et al., 2009), *ITGB8* rs10486391 (Su et al., 2010), *TNFA*‐238G>A rs361525 (Achrol et al., 2006, 2007)), and four variants in TGFβ modifier loci (*PTPN14* rs2936018, *USH2A* rs700024 (Benzinou et al., 2012), *ADAM17* rs12474540 and rs10495565 (Kawasaki et al., 2014)) using commercially available Taqman assays according to manufacturer's instructions (Applied Biosystems, Foster City, CA). Genotypes were scored by investigators blinded to phenotype. All genotype call rates were >95%. All variants were consistent with Hardy–Weinberg equilibrium (*p* > .05).

### 2.4 Statistical analysis

Genotypes were collapsed for analysis into risk genotype carriers versus noncarriers according to published associations with sporadic BAVM (Achrol et al., 2006, 2007; Hysi et al., 2007; Kim et al., 2009; Mikhak et al., 2011; Pawlikowska et al., 2004, 2005, 2006; Su et al., 2010; Weinsheimer et al., 2009). For the four TGFβ modifier variants, the published analysis (Benzinou et al., 2012; Kawasaki et al., 2014) was for familial transmission, which we cannot perform in our nonfamilial cohort, so we used an additive model for the minor (risk) allele, as this is the most general genetic model for a case–control analysis with the fewest additional assumptions. Association of genotype with presence of Any VM (pulmonary, liver or brain), pulmonary AVM, liver VM, brain VM, and ICH from brain VM was evaluated by multivariable logistic regression adjusted for age at last follow‐up, gender and family clustering. The same direction of effect (risk genotype) as previously published was required. The statistical significance threshold was adjusted for 11 variants tested and set at *p* = .0045. We also performed secondary analyses stratified by HHT mutation (*ENG* or *ACVRL1*) for all phenotypes except ICH (where the N of BAVM patients with known HHT mutations was too small to accommodate multivariate analyses for nine variants), and of the severe lung AVM phenotype (to evaluate the effect of a more stringent phenotype definition). One variant‐phenotype analysis could not be evaluated with multivariable logistic regression due to perfect prediction; we instead provide results from univariable exact logistic regression.

## 3 RESULTS

Table 1 shows demographic and clinical characteristics of the study cohort. Among 752 Caucasian HHT patients, 52% had pulmonary AVM, 20% had liver VM and 21% had brain VM. 20% (30/153) of brain VM cases had ICH (prior to enrollment or during follow‐up).

**Table 1:** Demographic characteristics and HHT phenotypes of study cohort

Characteristic	All Subjects	*ENG* mutation	*ACVRL1* mutation	*p*
Female sex	437/752 (58%)	143/240 (60%)	109/200 (55%)	.289
Age at last follow‐up (year)	47.4 ± 19.6	46.1 ± 19.0	49.0 ± 20.2	.121
HHT mutation:				n/a
*ENG*	240/455 (53%)	240/240 (100%)	n/a	
*ACVRL1*	200/455 (44%)	n/a	200/200 (100%)	
*SMAD4*	15/455 (3%)	n/a	n/a	
Any VM	526/728 (72%)	202/238 (85%)	101/194 (52%)	<.001
Lung AVM	378/730 (52%)	170/235 (72%)	37/195 (19%)	<.001
Liver VM	144/717 (20%)	24/230 (10%)	56/196 (29%)	<.001
Brain VM	156/752 (21%)	78/240 (33%)	22/200 (11%)	<.001
ICH from brain VM	30/153 (20%)	14/78 (18%)	5/22 (23%)	.759

Values are no. observed with the specified characteristic over the total no. of nonmissing observations or mean ± standard deviation.

*p*, comparison of *ACVRL1* and *ENG* subjects using Fisher's exact test or a two‐sample *t*‐test.

AVM, arteriovenous malformation; ICH, intracerebral hemorrhage; VM, vascular malformation.

None of the 11 variants was significantly associated with VM in any organ or with ICH (Table 2). We did not replicate the previously reported associations of four TGFB modifier variants (Benzinou et al., 2012; Kawasaki et al., 2014) with HHT lung AVM. There was a trend toward association of one of these variants, *USH2A* rs700024, with ICH from brain VM (OR = 2.77, 95% CI = 1.13–6.80, *p* = .026).

**Table 2:** Association of candidate variant genotype with HHT phenotypes

Phenotype	Polymorphism (risk genotypes)	*n*	OR	95% CI	*p*
Any VM	APOE ε2	713	0.83	0.52‐1.34	.452
	ANGPTL4 rs11672433 (AA or AG)	708	0.99	0.66‐1.49	.977
	EPHB4 rs314308 (AA or AG)	719	0.76	0.53‐1.08	.124
	IL1B‐31T>C, rs1143627 CC)	723	0.73	0.44‐1.21	.222
	IL6‐174G>C rs1800795 (GG)	714	0.87	0.61‐1.24	.443
	ITGB8 rs10486391 (AA)	725	0.83	0.58‐1.19	.311
	TNF‐238G>A rs361525 (AA or AG)	722	0.56	0.32‐1.00	.048
	ADAM17 rs10495565	726	1.04	0.81‐1.33	.775
	ADAM17 rs12474540	724	1.01	0.80‐1.28	.942
	PTPN14 rs2936018	717	0.96	0.71‐1.29	.784
	USH2A rs700024	708	1.12	0.72‐1.76	.613
Brain VM	APOE ε2	737	0.84	0.50‐1.40	.494
	ANGPTL4 rs11672433 (AA or AG)	732	0.78	0.50‐1.20	.256
	EPHB4 rs314308 (AA or AG)	743	1.28	0.89‐1.84	.181
	IL1B‐31T>C, rs1143627 CC)	746	1.00	0.57‐1.78	.988
	IL6‐174G>C rs1800795 (GG)	738	0.54	0.36‐0.83	.005
	ITGB8 rs10486391 (AA)	749	0.92	0.63‐1.37	.696
	TNF‐238G>A rs361525 (AA or AG)	746	1.01	0.59‐1.74	.959
	ADAM17 rs10495565	750	0.85	0.65‐1.10	.213
	ADAM17 rs12474540	748	0.79	0.61‐1.03	.085
	PTPN14 rs2936018	741	1.05	0.77‐1.44	.749
	USH2A rs700024	732	1.04	0.69‐1.55	.868
ICH from brain VM	APOE ε2	152	0.47	0.10‐2.35	.361
	ANGPTL4 rs11672433 (AA or AG)	151	1.89	0.78‐4.59	.158
	EPHB4 rs314308 (AA or AG)	153	1.85	0.69‐4.96	.222
	IL1B‐31T>C, rs1143627 CC)	152	1.22	0.37‐4.04	.750
	IL6‐174G>C rs1800795 (GG)	151	0.46	0.15‐1.44	.185
	ITGB8 rs10486391 (AA)	152	0.82	0.35‐1.96	.664
	TNF‐238G>A rs361525 (AA or AG)	151	0.93	0.24‐3.54	.915
	ADAM17 rs10495565	151	0.71	0.41‐1.21	.208
	ADAM17 rs12474540	153	0.70	0.42‐1.17	.171
	PTPN14 rs2936018	153	0.89	0.41‐1.93	.770
	USH2A rs700024	147	2.77	1.13‐6.80	.026
Liver VM	APOE ε2	702	0.95	0.53‐1.73	.876
	ANGPTL4 rs11672433 (AA or AG)	697	1.26	0.81‐1.96	.300
	EPHB4 rs314308 (AA or AG)	708	1.19	0.79‐1.79	.404
	IL1B‐31T>C, rs1143627 (CC)	712	0.40	0.19‐0.86	.019
	IL6‐174G>C rs1800795 (GG)	704	1.03	0.68‐1.56	.884
	ITGB8 rs10486391 (AA)	714	0.77	0.52‐1.15	.202
	TNF‐238G>A rs361525 (AA or AG)	711	0.78	0.41‐1.48	.445
	ADAM17 rs10495565	715	1.31	0.98‐1.77	.071
	ADAM17 rs12474540	714	1.42	1.08‐1.87	.013
	PTPN14 rs2936018	706	1.17	0.82‐1.67	.375
	USH2A rs700024	698	1.20	0.77‐1.85	.418
Lung AVM	APOE ε2	715	0.91	0.58‐1.43	.691
	ANGPTL4 rs11672433 (AA or AG)	710	0.88	0.62‐1.25	.471
	EPHB4 rs314308 (AA or AG)	721	0.78	0.57‐1.07	.120
	IL1B‐31T>C, rs1143627 CC)	724	0.98	0.61‐1.57	.938
	IL6‐174G>C rs1800795 (GG)	716	1.10	0.80‐1.51	.555
	ITGB8 rs10486391 (AA)	727	0.70	0.52‐0.96	.027
	TNF‐238G>A rs361525 (AA or AG)	724	0.54	0.33‐0.89	.015
	ADAM17 rs10495565	728	0.98	0.79‐1.22	.879
	ADAM17 rs12474540	726	0.92	0.75‐1.14	.462
	PTPN14 rs2936018	719	1.00	0.77‐1.31	.994
	USH2A rs700024	710	1.03	0.72‐1.46	.892

*p*, multivariable regression adjusted for gender, age at last follow‐up and family clustering.

AVM, arteriovenous malformation; ICH, intracerebral hemorrhage; VM, vascular malformation.

In secondary analysis, we detected no statistically significant associations between the severe lung AVM subphenotype (100 out of 664 patients (15%) with data available) and the four TGFB modifier loci SNPs (ORs between 0.87 and 0.99, all *p* > .33). In analysis stratified by *ENG* or *ACVRL1* HHT mutation, among *ENG* mutation carriers only, liver VM were associated with *ADAM17* rs12474540 (OR = 2.82, 95% CI = 1.37–4.30, *p* = .002, Table S1). There was also a trend toward association of liver VM with *ADAM17* rs10495565 (OR = 2.28, 95% CI = 1.23–4.20, *p* = .009), and with *PTPN14* rs2936018 (OR = 2.82, 95% CI = 1.31–6.08), *p* = .008) (Table S1).

## 4 DISCUSSION

We did not replicate the previously reported associations between four TGFB modifier loci variants with HHT lung AVM (Benzinou et al., 2012; Kawasaki et al., 2014) in the overall BVMC cohort. In analysis stratified by HHT gene, among *ENG* mutation carriers only, there was a significant association of one of the four variants, *ADAM17* rs12474540, and a trend toward association of two other TGFB modifier SNPs with liver (but not lung) VM. There was a trend in the same direction in the overall cohort. The lung AVM associations originally reported were also stronger among *ENG* mutation carriers (Kawasaki et al., 2014). There was also a trend toward association of another of these variants, *USH2A* rs700024, with ICH from brain VM. These findings may indicate that the TGFB modifier loci variants are associated with multiple HHT phenotypes, but a larger cohort will be required to confirm this. It is not clear why we do not detect a lung AVM association in our cohort, and do not replicate the original finding (Kawasaki et al., 2014). To check for a possible effect of different lung AVM ascertainment on the genetic association results, we defined a severe lung AVM phenotype closer to the lung AVM phenotype definition used in the cohort studied in the original report (Letteboer et al., 2015; Pawlikowska et al., 2015), but did not detect statistically significant associations with any of the 4 TGFB modifier variants. The disparity in genetic association results may also be influenced by methodological differences: the published analysis of lung AVM was performed in families, testing for transmission of risk alleles; we performed a case–control association analysis in a large cohort of mostly unrelated subjects. Another difference between our cohorts was that the BVMC targeted enrollment of 25% brain VM positive patients, resulting in cohort with 21% brain VM positive patients. This proportion is within the upper end of the range of prevalence of brain VM positive patients reported by other cohorts (5%–23% (Akers et al., 2013)), so we do not expect it to significantly influence the results of our association analysis, however it does represent a divergence from completely unbiased enrollment. Finally, our cohort was North American Caucasians, the original findings were in Dutch and French Caucasians (Benzinou et al., 2012; Kawasaki et al., 2014). It is possible for population stratification even among/within Caucasian cohorts to influence genetic association results.

We did not detect significant associations between any of the 7 variants previously reported in sporadic BAVM or ICH with HHT brain VM or ICH. Possible explanations include different genetic influences on sporadic and HHT brain AVM, that the original findings (from small cohorts and unreplicated) were false positives, and a false negative, due to lack of power to detect small effects in our cohort. In the case of *ACVRL1* c.314‐35A>G, the sporadic brain AVM‐associated variant that we found to also be associated with VM in HHT (Pawlikowska et al., 2015), the original finding had been replicated in multiple sporadic brain AVM cohorts, and the variant was in an HHT gene. The BVMC cohort of 752 subjects is substantial for a rare disease such as HHT, but may not be sufficient to detect associations of small effect size. We did observe several associations with the same direction of effect as in previous studies; a larger cohort is required to validate these associations. The BVMC is recruiting a second cohort of 800 HHT patients.

Our findings suggest the effects of genetic modifier variants are complex, but given the functional data from animal and in vitro models for the involvement of ADAM17 and PTPN14 in HHT biology (Benzinou et al., 2012; Kawasaki et al., 2014), it is of interest to revisit these in larger cohorts. Identification and validation of genetic modifiers could inform prognosis and guide clinical management of HHT patients and provide potentially useful stratifier biomarkers for clinical trials.

## CONFLICT OF INTEREST

None declared.

## APPENDIX 1

### 1.1

Brain Vascular Malformation Consortium HHT Investigator Group: Murali Chakinala, Marianne Clancy, Marie E. Faughnan, James R. Gossage, Katharine Henderson, Steven W. Hetts, Vivek Iyer, Raj Kasthuri, Helen Kim, Timo Krings, Michael T. Lawton, Doris Lin, Johannes Jurgen Mager, Douglas Marchuk, Justin P. McWilliams, Jamie McDonald, Ludmila Pawlikowska, Jeffrey Pollak, Felix Ratjen, Karen Swanson, Karel terBrugge, Dilini Vethanayagam, Andrew White, Pearce Wilcox.
