# Supplementary material for: Association of common candidate variants with vascular malformations and intracranial hemorrhage in hereditary hemorrhagic telangiectasia
Source: Mol Genet Genomic Med. 2018 Mar 6;6(3):350–6. doi: 10.1002/mgg3.377 (PMC6014448; doi:10.1002/mgg3.377)
Supplement: Supplementary file 1 [file MGG3-6-350-s001.docx]

**SUPPLEMENTAL TABLE I. Association analysis stratified by HHT mutation.**

|  |  | ***ENG* (HHT1) subjects** | | | | ***ACVRL1* (HHT2) subjects** | | | |
| --- | --- | --- | --- | --- | --- | --- | --- | --- | --- |
| **Phenotype** | **Polymorphism**  **(risk genotypes)** | **n** | **OR** | **95% CI** | **P** | **n** | **OR** | **95% CI** | **P** |
| **Any VM** | APOE ε2 | 230 | 0.49 | 0.20 - 1.21 | 0.123 | 191 | 0.51 | 0.19 - 1.35 | 0.176 |
|  | ANGPTL4 rs11672433 (AA or AG) | 229 | 2.01 | 0.72 - 5.62 | 0.182 | 189 | 0.98 | 0.45 - 2.16 | 0.966 |
|  | EPHB4 rs314308 (AA or AG) | 235 | 0.45 | 0.20 - 1.02 | 0.202 | 192 | 0.95 | 0.52 - 1.76 | 0.875 |
|  | IL1B-31T>C, rs1143627 (CC) | 235 | 1.19 | 0.39 - 3.62 | 0.755 | 193 | 0.68 | 0.27 - 1.76 | 0.429 |
|  | IL6-174G>C rs1800795 (GG) | 232 | 0.69 | 0.31 - 1.57 | 0.378 | 191 | 1.06 | 0.56 - 2.01 | 0.858 |
|  | ITGB8 rs10486391 (AA) | 236 | 0.70 | 0.31 - 1.59 | 0.399 | 194 | 0.92 | 0.49 - 1.75 | 0.799 |
|  | TNF-238G>A rs361525 (AA or AG) | 235 | 1.23 | 0.36 - 4.20 | 0.746 | 192 | 0.43 | 0.12 - 1.57 | 0.201 |
|  | ADAM17 rs10495565 | 237 | 1.06 | 0.62 - 1.83 | 0.823 | 194 | 0.81 | 0.50 - 1.33 | 0.411 |
|  | ADAM17 rs12474540 | 237 | 0.99 | 0.56 - 1.73 | 0.960 | 193 | 0.89 | 0.60 - 1.33 | 0.578 |
|  | PTPN14 rs2936018 | 235 | 0.87 | 0.47 - 1.60 | 0.650 | 190 | 1.41 | 0.79 - 2.54 | 0.245 |
|  | USH2A rs700024 | 228 | 1.38 | 0.58 - 3.27 | 0.469 | 190 | 1.27 | 0.60 - 2.68 | 0.527 |
| **Brain VM** | APOE ε2 | 232 | 1.21 | 0.61 - 2.42 | 0.581 | 197 | 0.19 | 0 - 1.13* | 0.073 |
|  | ANGPTL4 rs11672433 (AA or AG) | 231 | 0.90 | 0.46 - 1.76 | 0.757 | 195 | 0.48 | 0.15 - 1.52 | 0.212 |
|  | EPHB4 rs314308 (AA or AG) | 237 | 1.04 | 0.60 - 1.81 | 0.893 | 198 | 1.92 | 0.77 – 4.77 | 0.160 |
|  | IL1B-31T>C, rs1143627 (CC) | 237 | 1.13 | 0.50 - 2.54 | 0.764 | 199 | 1.92 | 0.47 - 7.88 | 0.364 |
|  | IL6-174G>C rs1800795 (GG) | 234 | 0.85 | 0.46 - 1.56 | 0.598 | 197 | 0.54 | 0.18 - 1.59 | 0.261 |
|  | ITGB8 rs10486391 (AA) | 238 | 1.20 | 0.66 - 2.17 | 0.557 | 200 | 0.92 | 0.35 - 2.42 | 0.859 |
|  | TNF-238G>A rs361525 (AA or AG) | 237 | 1.11 | 0.46 - 2.67 | 0.812 | 198 | 0.76 | 0.18 - 3.26 | 0.709 |
|  | ADAM17 rs10495565 | 239 | 0.87 | 0.59 - 1.29 | 0.499 | 200 | 0.66 | 0.34 - 1.26 | 0.208 |
|  | ADAM17 rs12474540 | 239 | 0.73 | 0.51 - 1.06 | 0.101 | 199 | 0.87 | 0.45 - 1.67 | 0.676 |
|  | PTPN14 rs2936018 | 237 | 1.09 | 0.68 - 1.77 | 0.715 | 196 | 1.39 | 0.65 - 2.94 | 0.397 |
|  | USH2A rs700024 | 230 | 1.61 | 0.87 - 2.96 | 0.130 | 196 | 0.27 | 0.04 - 1.98 | 0.198 |
| **Liver VM** | APOE ε2 | 222 | 1.07 | 0.28 - 4.10 | 0.922 | 193 | 0.49 | 0.14 - 1.67 | 0.251 |
|  | ANGPTL4 rs11672433 (AA or AG) | 221 | 1.34 | 0.53 - 3.41 | 0.537 | 191 | 1.26 | 0.53 - 2.96 | 0.601 |
|  | EPHB4 rs314308 (AA or AG) | 227 | 1.92 | 0.71 – 5.18 | 0.201 | 194 | 0.58 | 0.29 - 1.14 | 0.111 |
|  | IL1B-31T>C, rs1143627 (CC) | 227 | 0.62 | 0.13 - 2.87 | 0.537 | 195 | 0.99 | 0.30 - 3.33 | 0.991 |
|  | IL6-174G>C rs1800795 (GG) | 225 | 1.81 | 0.76 - 4.36 | 0.183 | 193 | 0.79 | 0.37 - 1.69 | 0.537 |
|  | ITGB8 rs10486391 (AA) | 228 | 0.54 | 0.21 - 1.33 | 0.179 | 196 | 1.06 | 0.52 - 2.18 | 0.872 |
|  | TNF-238G>A rs361525 (AA or AG) | 227 | 3.02 | 0.98 - 9.28 | 0.054 | 194 | 0.28 | 0.05 - 1.41 | 0.122 |
|  | ADAM17 rs10495565 | 229 | 2.28 | 1.23 - 4.20 | 0.009 | 196 | 0.93 | 0.53 - 1.63 | 0.795 |
|  | **ADAM17 rs12474540** | **229** | **2.43** | **1.37 - 4.30** | **0.002** | 195 | 1.19 | 0.73 - 1.95 | 0.476 |
|  | PTPN14 rs2936018 | 227 | 2.82 | 1.31 - 6.08 | 0.008 | 192 | 0.67 | 0.33 - 1.35 | 0.260 |
|  | USH2A rs700024 | 221 | 2.05 | 0.79 - 5.31 | 0.140 | 192 | 0.73 | 0.30 - 1.76 | 0.480 |
| **Lung AVM** | APOE ε2 | 227 | 0.42 | 0.17 - 1.03 | 0.058 | 192 | 1.37 | 0.51 - 3.72 | 0.536 |
|  | ANGPTL4 rs11672433 (AA or AG) | 226 | 1.55 | 0.69 - 3.50 | 0.291 | 190 | 0.82 | 0.31 - 2.14 | 0.684 |
|  | EPHB4 rs314308 (AA or AG) | 232 | 0.54 | 0.28 - 1.04 | 0.066 | 193 | 1.28 | 0.54 – 3.04 | 0.577 |
|  | IL1B-31T>C, rs1143627 (CC) | 232 | 1.10 | 0.46 - 2.65 | 0.829 | 194 | 0.49 | 0.11 - 2.27 | 0.362 |
|  | IL6-174G>C rs1800795 (GG) | 229 | 1.03 | 0.52 - 2.02 | 0.943 | 192 | 1.11 | 0.53 - 2.29 | 0.787 |
|  | ITGB8 rs10486391 (AA) | 233 | 0.57 | 0.31 - 1.05 | 0.070 | 195 | 0.92 | 0.39 - 2.14 | 0.844 |
|  | TNF-238G>A rs361525 (AA or AG) | 232 | 0.69 | 0.30 - 1.59 | 0.378 | 193 | 0.48 | 0.09 - 2.48 | 0.383 |
|  | ADAM17 rs10495565 | 234 | 0.90 | 0.59 - 1.37 | 0.628 | 195 | 1.29 | 0.78 - 2.11 | 0.319 |
|  | ADAM17 rs12474540 | 234 | 0.98 | 0.63 - 1.50 | 0.913 | 194 | 1.03 | 0.63 - 1.69 | 0.902 |
|  | PTPN14 rs2936018 | 232 | 1.00 | 0.60 - 1.69 | 0.986 | 191 | 1.52 | 0.76 - 3.05 | 0.236 |
|  | USH2A rs700024 | 225 | 1.23 | 0.64 - 2.37 | 0.542 | 191 | 1.96 | 0.83 - 4.62 | 0.126 |

P, multivariable regression adjusted for gender, age at last follow-up and family, except *one-sided confidence interval from exact logistic regression analysis. There were not enough subjects to perform stratified analysis of brain VM ICH. VM, vascular malformation, AVM, arteriovenous malformation, ICH, intracerebral hemorrhage.
